# Detecting Bioterror Attack

**DOI:** 10.3201/eid1008.031044

**Published:** 2004-08

**Authors:** Dominique Bicout

**Affiliations:** * Ecole National Veterinaire Lyon, Marcy-l'Etoile, France

**Keywords:** modeling, epidemic processes, bioterror attack

**To the Editor:** In a recent article (*1*), Kaplan et al. addressed the problems in detecting a bioterror attack from blood-donor screening. The main point of this comment is the "early approximation" used by Kaplan et al. to derive the probability of detecting an attack. The simplification used by Kaplan et al. leads to a probability that does not account for the size of the exposed population and can lead to incorrect results and misinterpretations.

Consider a single bioterror attack that infects a proportion p of an exposed population of size N at time τ = 0, such that the initial number of infected is *I_0_*= Np. The quantity of interest is the probability *D*(τ) of finding at least one positive blood donation and detecting the attack within time τ. For attacks conducted with contagious agents that could lead to an epidemic, Kaplan et al. used the early approximation solution of the classic epidemic models (*2*) to describe the progression of the number of infected persons. Consequently, the resulting probability of attack detection [noted *D*_es_(τ)] is dependent only upon the initial size of the release I_0_ , the basic reproductive number *R*_0_ (the mean number of secondary cases per initial index case), and other variables (the blood screening window ω, the mean number k of blood donations per person and per unit of time, and the mean duration of infectiousness 1/r) (Appendix). Early approximation can lead to unreliable results because it is valid only at earlier stages of the epidemics and in the limit where the proportion p of initially infected is much smaller than the intrinsic steady proportion (*R*_0_ – 1) / *R*_0_ of the epidemics (Appendix). Relaxing this approximation and using the full solution for the progression of the number of infected persons leads to the probability *D*(τ)that takes into account the size of the exposed population (Appendix). The latter is important because, in contrast to *D*_es_(τ)that leads to the same conclusion, *D*(τ) indicates that the probabilities of detecting an attack within two exposed populations of different sizes, but with the same numbers of initially infected, are not identical. As illustrated in the Figure, when the other variables are fixed, *D*(τ)decreases as the proportion p of initially infected increases because the epidemic size decreases as p approaches the threshold (*R*_0_ – 1) / *R*_0_ . These subtleties of a simple epidemic model are even less reliable when using the blood screening to detect a bioterror attack with agents that cause diseases of very short incubation period.

**Figure:**
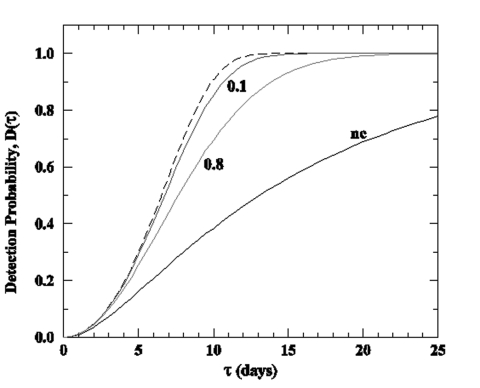
Probability of attack detection delay for a contagious agent. Dashed line represents the early approximation D_es_(τ), solid lines the full solution (where the numbers represent the fraction p of the population initially infected), and the symbol "nc" stands for noncontagious agent (*R*_0_=0). The parameters are as follows: blood donation rate k = 0.05 per person per year, screening mean window period ω = 3 days, mean duration of infectiousness 1/r = 14 days, basic reproductive number *R*_0_=5, and the initial attack size Np = 500. Note that the exposed populations are therefore 5,000 and 625 for p = 0.1 and p= (*R_0_*-1) / *R_0_* = 0.8 , respectively.

Nonetheless, detecting a bioterror attack is very similar to detecting the response of pathogen-specific immunoglobulin M antibodies (as an indicator of recent contact of hosts with pathogens) within a population of hosts by using serologic surveys. Therefore, the reasoning developed for a bioterror attack can be extended and applied to detect and time the invasion or early circulation of certain pathogens within a population. In that perspective, it might be useful to develop an analysis that includes more details of the epidemic progression within this framework.

## Appendix

Following Kaplan et al. (*1*), the probability *D*(τ) of finding at least one positive blood donation and detecting the attack within time τ**,** after a single bioterror attack initially infecting a proportion *P* of an exposed population of size *N*, is given by


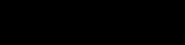
, where *_K_* is the mean number of blood donations per person and per unit of time and 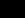 is the probability that, within the blood screening window period of ∞ days, a randomly selected member of the population would test positive *t* days after the attack, _
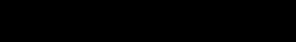
_. As cited (*1*), the progression of the number of infected persons *I*(t) is described using the differential equation (*2*), *dI / dt = (rR_0_ / N)(N-I) – rI* , with the initial condition *I(_0_) = I_0_ = Np*. From this, we have,



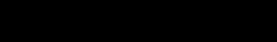




[1]

When *R*_0_ > 1, this logistic function increases, remains constant or decreases from the initial value *I(_0_) = I_0_* towards the steady state *I*(*t* →∞) = I_∞_ = (*R*_0_ – 1) *N* / *R*_0_ for

*I_0_ < 1_∞_, I_0_ - I_∞_, or I_0_> I_∞_*, respectively. In particular, in the limit of *_P_* << (*R_0_* – 1) / *R*_0_ , this expression reduces to the early approximation solution, *I_es_ (t) – I_0_* exp {-(*R*_0_ – 1)*rt* }, and the resulting probability D_es_(τ)of attack detection is instead,

D_es_(τ) = 1 – exp{-1I_0_[*a*ƒ(τ /∞) − *b*ƒ[(*R*_0_ – 1) rτ ]]}

[2]

where the function, ƒ(x) = x – 1 + exp (-x), and the constants are, *a* = *k*∞ [1 - r ∞(2*R*_0_ – 1)] / [1-r∞(*R*_0_-1)] and *b= kR_0_ /* {*r* (*2R_0_ – 1*)*^2^*{*1-r∞*(*R_0_-*1)]}. This D_es_(τ) increases when any of the parameters increase.
