# Supplementary material for: Detecting Bioterror Attack
Source: Emerg Infect Dis. 2004 Aug;10(8):1504–5. doi: 10.3201/eid1008.031044 (PMC3320411; doi:10.3201/eid1008.031044)
Supplement: Related texts

# Reply: Detecting Bioterror Attack

**DOI:** 10.3201/eid1008.040291

**Authors:** Edward H. Kaplan, Lawrence M. Wein

**Affiliations:** * Yale School of Management , New Haven, Connecticut, USA;; † Stanford University, Stanford, California, USA

**In Reply:** As stated and argued throughout our article (*1*), we conducted a best-case analysis under assumptions that favored blood-donor screening to detect bioterror attacks; if such an analysis fails to justify donor screening, no analysis will. Bicout (*2*) is concerned about our assumption of exponential infection growth after attack; however, this assumption was one of several we made deliberately as part of our best-case scenario (*1*).

Bicout's calculations actually reinforce rather than refute our analysis. By relaxing our assumption of exponential infection growth and using the well-known logistic solution to the basic epidemic model (equation 1 in Bicout's letter), Bicout shows that more time is required to detect a bioterror attack than when exponential infection growth is assumed (Figure accompanying Bicout's letter). The number of persons infected over time under the logistic model will be fewer than the number of persons infected if exponential growth is assumed; therefore, screening blood donors to detect a bioterror attack is even less attractive than using our best-case assumptions. The take-home message from our article was and is: It makes little sense to screen blood donors to detect a bioterror attack.

## References

[1] Kaplan EH, Patton CA, FitzGerald WP, Wein LM. Detecting bioterror attacks by screening blood donors: a best-case analysis. Emerg Infect Dis. 2003;9:909–14.1296748610.3201/eid0908.030079PMC3020608

[2] Bicout DJ. Detecting bioterror attack [letter]. Emerg Infect Dis. 2004;10:1504b–5. 10.3201/eid1008.03104415503398PMC3320411
